# A Baseline Model For Estimating the Risk of Gas Embolism in Sea Turtles During Routine Dives

**DOI:** 10.3389/fphys.2021.678555

**Published:** 2021-09-01

**Authors:** Nathan J. Robinson, Daniel García-Párraga, Brian A. Stacy, Alexander M. Costidis, Gabriela S. Blanco, Chelsea E. Clyde-Brockway, Heather L. Haas, Craig A. Harms, Samir H. Patel, Nicole I. Stacy, Andreas Fahlman

**Affiliations:** ^1^Department of Research, Fundación Oceanogràfic de la Comunidad Valenciana, Valencia, Spain; ^2^National Oceanic and Atmospheric Administration, National Marine Fisheries Service, Office of Protected Resources, University of Florida (duty station), Washington, DC, United States; ^3^Virginia Aquarium and Marine Science Center, Virginia Beach, VA, United States; ^4^Instituto de Biología de Organismos Marinos (IBIOMAR-CCT CONICET-CENPAT), Puerto Madryn, Argentina; ^5^The Leatherback Trust, Fort Wayne, IN, United States; ^6^Northeast Fisheries Science Center, National Marine Fisheries Service, National Oceanic and Atmospheric Administration, Woods Hole, MA, United States; ^7^Department of Clinical Sciences and Center for Marine Sciences and Technology, North Carolina State University, Raleigh, NC, United States; ^8^Coonamessett Farm Foundation, East Falmouth, MA, United States; ^9^Department of Comparative, Diagnostic, and Population Medicine, College of Veterinary Medicine, University of Florida, Gainesville, FL, United States; ^10^Global Diving Research, Inc., Ottawa, ON, Canada

**Keywords:** Physiology, ecological modeling, fisheries, decompression sickness, conservation, dive behavior

## Abstract

Sea turtles, like other air-breathing diving vertebrates, commonly experience significant gas embolism (GE) when incidentally caught at depth in fishing gear and brought to the surface. To better understand why sea turtles develop GE, we built a mathematical model to estimate partial pressures of N_2_ (PN_2_), O_2_ (PO_2_), and CO_2_ (PCO_2_) in the major body-compartments of diving loggerheads (*Caretta caretta*), leatherbacks (*Dermochelys coriacea*), and green turtles (*Chelonia mydas*). This model was adapted from a published model for estimating gas dynamics in marine mammals and penguins. To parameterize the sea turtle model, we used values gleaned from previously published literature and 22 necropsies. Next, we applied this model to data collected from free-roaming individuals of the three study species. Finally, we varied body-condition and cardiac output within the model to see how these factors affected the risk of GE. Our model suggests that cardiac output likely plays a significant role in the modulation of GE, especially in the deeper diving leatherback turtles. This baseline model also indicates that even during routine diving behavior, sea turtles are at high risk of GE. This likely means that turtles have additional behavioral, anatomical, and/or physiologic adaptions that serve to reduce the probability of GE but were not incorporated in this model. Identifying these adaptations and incorporating them into future iterations of this model will further reveal the factors driving GE in sea turtles.

## Introduction

Until the early 2000s, it was assumed that most air-breathing marine vertebrates were not susceptible to gas embolism (GE) or its associated diseases (Jepson et al., 2003). However, several studies have now provided direct evidence of GE in cetaceans, pinnipeds, and sea turtles (Van Bonn et al., 2011, 2013; Dennison et al., 2012a,b; Crespo-Picazo et al., 2020). Under natural conditions, most air-breathing marine vertebrates likely manage the risk of GE through a combination of behavioral, anatomical, and physiological adaptations (García-Párraga et al., 2018b; Fahlman et al., 2020, 2021). Yet there appears to be significant increase in the potential for GE if animals make abnormally rapid ascents after diving (Fahlman et al., 2021). This could be caused by animals being caught incidentally in fishing gear (Fahlman et al., 2017) or if they are disturbed by acoustic sources such as mid-frequency sonar (Tal et al., 2015). Severe cases of GE can even lead to immediate or delayed mortality after several days (Parga et al., 2020). Consequently, GE likely poses an underestimated threat to marine animals that are subject to high levels of fisheries bycatch such as sea turtles (Wallace et al., 2010, 2013).

The formation of gas emboli is driven by changes in gas dynamics associated with the variable hydrostatic pressure that an animal experiences while diving. During the descent, hydrostatic pressure increases in proportion to depth. For air-breathing vertebrates, the increased pulmonary gas tensions cause the gaseous N_2_, O_2_, and CO_2_ in the lungs to dissolve into solution within the pulmonary circulatory system (Fahlman et al., 2006, 2021). The gases are then transported *via* the vascular system throughout the various tissues of the body (Fahlman et al., 2006, 2009). The amount of gas that dissolves into the tissues during a dive is, therefore, influenced by the level of gas exchange in the lungs, cardiac output (heart rate and stroke volume), blood flow distribution, and tissue perfusion (Fahlman et al., 2006, 2009, 2018). Initially, the rate at which gas diffuses across the lungs increases with depth as higher hydrostatic pressures increase the quantity of gas forced into solution in the blood (Scholander, 1940; Berkson, 1967). However, if hydrostatic pressures continue to increase, the lung alveoli (or ediculi and faveoli in reptiles) compress and eventually collapse (Bostrom et al., 2008; Fahlman et al., 2009; Mcdonald and Ponganis, 2012). When all the alveoli have collapsed, gas exchange in the lungs ceases. Many diving animals also further reduce the rate at which gases dissolve in the their tissues through changes in circulation and perfusion (Fahlman et al., 2006). Specifically, some animals reduce cardiac output and blood flow to the lungs while simultaneously redistributing blood flow to O_2_-demanding tissues (Scholander, 1940).

On ascent, diminishing hydrostatic pressures decrease gas tensions and so the gases come out of solution. If this process occurs faster than the animal can transfer the excess blood gas to the lungs, intravascular gas bubbles will form (Fahlman, 2017; Fahlman et al., 2021). While minor GE can be tolerated, serious cases can damage neural and more vascularized tissues. In turn, this can lead to impaired motor skills, loss of consciousness, paralysis, and even death (van Hulst et al., 2003; Dennison et al., 2012b; García-Párraga et al., 2014).

Several recent studies have confirmed that GE in sea turtles is a wide-spread phenomenon associated with fisheries bycatch (García-Párraga et al., 2014; Crespo-Picazo et al., 2020; Parga et al., 2020) and can also occur in other interactions with submerged gear such as Hopper dredges (Harms et al., 2020). It has also been shown that the depth of capture is a key component in determining the likelihood of GE. Loggerhead turtles (*Caretta caretta*) caught by Mediterranean trawlers and gillnets at depths exceeding 65 m were 50% more likely to suffer from fatal GE than those caught at shallower depths (Fahlman et al., 2017). To understand why turtles are susceptible to fisheries-induced GE, we must first understand tissue gas dynamics during normal diving behavior. For example, it is possible that sea turtles live with elevated tissue N_2_ levels, comparable to that of a saturation diver (Brubakk et al., 2011). If so, even minor changes to routine diving patterns or disruption to those anatomical or physiological adaptations governing cardiac output and blood flow could lead to GE.

Our goal in this study was to develop a baseline model to estimate the risk of GE formation in sea turtles. To build this model, we built upon a published model for estimating gas tissue N_2_, O_2_, and CO_2_ tensions of marine mammals and penguins (Fahlman et al., 2006, 2007, 2009, 2018, 2021) and adapted it for use with loggerhead turtles, green turtles (*Chelonia mydas*), and leatherback turtles (*Dermochelys coriacea*). While there are many anatomical and physiological differences between marine mammals/penguins and sea turtles, building off these earlier models provided us with an initial framework for modeling gas dynamics that had been previously validated for use with air-breathing vertebrates. We parameterized the model based on estimates of key tissue compartment volumes in sea turtles derived from necropsies and computed tomography. We also gleaned other parameters on sea turtle metabolic rate and cardiac output data from the published literature. Next, we used this model to estimate partial pressures of N_2_ (PN_2_), O_2_ (PO_2_), and CO_2_ (PCO_2_) in various body compartments during routine dive patterns for each turtle species. Finally, we used this model to investigate whether (1) the percentage of body-fat (as a proxy for body-condition) and (2) cardiac output influenced the risk of GE. We chose to investigate body-fat as it changes the dynamics of the body N_2_ stores and has been considered as a potential risk variable in GE in humans (Lam and Yau, 1989; Schellart et al., 2012) and cetaceans (Fahlman et al., 2021). In addition, the volume of body fat is strongly influenced by reproduction in sea turtles (Davenport et al., 2011). We chose to investigate cardiac output as modeling efforts for marine mammals have shown this to be a key component influencing the risk of GE (Fahlman et al., 2006, 2009, 2021; Hooker et al., 2009).

## Materials and Methods

### Data Sets

We analyzed time-depth recorder (TDR) datasets from three loggerhead, six leatherback, and four green turtles (Table 1). The TDRs deployed on loggerhead turtles were Satellite Relay Data Loggers (SRDL–Sea Mammal Research Unit, United Kingdom), while MK10 tags (Wildlife Computers, United States) were used for both leatherback and green turtles. The loggerhead TDRs were attached to the top of the carapace using epoxy (for details, see Patel et al., 2018). For leatherback and green turtles, the TDRs were tethered to the posterior position of the carapace (for details, see Robinson et al., 2016). The loggerhead turtles were sub-adults sampled at foraging areas in the Mid-Atlantic Bight, while the leatherback and green turtles were inter-nesting females sampled on the east coast of South Africa and the northwest coast of Costa Rica, respectively (Table 1). The TDRs recorded depth to within 1 m accuracy at time-intervals of either 4 or 10 s. We standardized the sampling interval for all TDRs by interpolating the data to 1 s intervals.

**Table 1:** Time-depth data used in gas dynamics model. Data were analyzed from three loggerhead, six leatherback, and four green turtles.

Species	*ID*	*Body mass* (kg)	Life-stage/Sex	Sampling frequency(s)	Recording duration (days)	Location
Leatherback	Dc1	334^1^	Adult female (gravid)	10	21	SA
Leatherback	Dc2	356^1^	Adult female (gravid)	10	11	SA
Leatherback	Dc3	392^1^	Adult female (gravid)	10	10	SA
Leatherback	Dc4	217^1^	Adult female (gravid)	10	34	SA
Leatherback	Dc5	371^1^	Adult female (gravid)	10	21	SA
Leatherback	Dc6	360^1^	Adult female (gravid)	10	10	SA
Loggerhead	Cc1	44	Subadult, Sex Unknown	4	476(^*^139)	MAB
Loggerhead	Cc2	89	Subadult, Sex Unknown	4	187(^*^46)	MAB
Loggerhead	Cc3	60^2^	Subadult, Sex Unknown	4	401(^*^116)	MAB
Green	Cm1	71	Adult female (gravid)	10	54	CR
Green	Cm2	81^3^	Adult female (gravid)	10	41	CR
Green	Cm3	65^3^	Adult female (gravid)	10	22	CR
Green	Cm4	70^3^	Adult female (gravid)	10	11	CR

*For each individual, we note the species, ID, body-mass, life-stage/sex, time-depth sampling frequency, recording duration, and the sampling location (SA- iSimangaliso Wetland Park, South Africa; MAB- Mid-Atlantic bight; CR- Playa Cabuyal, Costa Rica). To estimate body-mass for leatherback turtles, we first converted Curved Carapace Length to Straight Carapace Length following an equation in (Blanco et al., 2013) and then from Straight Carapace Length to body-mass following an equation in Georges and Fosette (2006). Subscript numbers refer to reference for the equations used to convert Curved Carapace Length to body-mass equations:*

1 (Jean-Yves and Sabrina, 2006).

2 In-situ weights collected.

3 (Bjorndal and Bolten, 1988).

* number of days used for modeling.

TDR datasets ranged in duration 187–476 days for loggerhead turtles, 10–34 days for leatherback turtles, and 11–54 days for green turtles. As loggerheads are known to modify their diving behavior in response to seasonal variation in water temperatures (Iverson et al., 2019) and conduct extended “hibernation” dives during winter (Hochscheid et al., 2005), we only analyzed loggerhead data from the first 46 to 139 days (Table 1). This ensured that all data were collected during the summer months (May to November) when tag-derived sea surface temperature values were between 19 and 26°C. In contrast, tracking duration was short enough for leatherback and green turtles to assume that there were no significant changes in diving behavior due to seasonal factors within the available datasets. Moreover, all the leatherback and green turtles’ data were collected from tropical or sub-tropical habitats, where the sea surface temperature values only varied between 24 and 29°C. The loggerhead TDR data have been previously published in Patel et al. (2018). The leatherback and green turtle data have been published in Blanco et al. (2013), Robinson et al. (2017), and Clyde-Brockway et al. (2019).

### Model

To estimate gas dynamics in diving sea turtles, we adapted a model that was developed for estimating blood and gas tissue N_2_, O_2_, and CO_2_ tensions in marine mammals and penguins (Fahlman et al., 2006, 2007, 2009, 2018, 2021). In the model, gas exchange first occurs between the respiratory system and arterial blood, and then between the arterial blood and four compartments: (1) the brain; (2) fat and bone; (3) the central circulatory system, which included the heart, kidney, liver, and digestive tract but not blood; and (4) muscle, which included muscle, skin, connective tissue, and all other tissues and organs that were not included in the other compartments. Gas exchange next occurs between the four compartments and the mixed venous blood, and finally between the mixed venous blood and the respiratory system.

Gas exchange was driven by partial pressure/tension gradients as detailed in Fahlman et al. (2006), and a physiologic pulmonary shunt was allowed at increasing pressures due to alveolar compression (Bostrom et al., 2008; Fahlman et al., 2009). While reptilian lungs have faveoli or ediculi instead of the alveoli in mammals, the similarities in their structures mean that they likely respond similarly under pressure differentials. For ease of use, we hereafter refer to these differing features under the singular term faveoli. It should also be noted that sea turtles have the capacity to perform a right-to-left intracardiac shunt. As the role of this cardiac shunt in regulating N_2_ is unknown in aquatic reptiles (Burggren et al., 2020), we therefore adjusted the parameters for the modeled pulmonary shunt, so that it mirrored the arterial PN_2_ levels that previously reported for a sea turtles during forced submergence (Berkson, 1967).

The model assumed that breathing began the instant the animal reached the surface (i.e., a depth of 0 m). However, due to a combination of measurement error, the use of tethered tags on both leatherback and green turtles, and the original sampling frequency of the TDRs, not all breathing events were associated with a depth measurement of 0. While many studies address this issue by defining a dive as the period when a turtle descends beyond a pre-determined depth (e.g., 2 m), we chose to not use this dive definition as previous models have shown the risk of GE increases rapidly as a diver approaches the surface (Fahlman et al., 2018). Instead, we subtracted 2 m from all depth values. While this ensured that all breathing events were associated with a measurement of 0 depth, we acknowledge that this also slightly reduced the maximum depth of the dives.

When breathing, the arterial blood gas tensions of N_2_, O_2_, and CO_2_ were set to 0.741 atmospheres absolute (ATA), 0.164 ATA, and 0.033 ATA, respectively, as have been previously reported for loggerhead turtles (Lutcavage and Lutz, 1991) with water vapor being 0.062 ATA (Lutcavage and Lutz, 1991; Fahlman et al., 2008). We also assumed that arterial blood gas tensions while breathing were equal to the partial pressures in the faveoli, and that all CO_2_ that exchanged for O_2_ remained in gas phase in the lungs and did not dissolve into the lung parenchyma (Fahlman et al., 2006).

### Lung Compression and Pulmonary Shunt

We used the model published by Bostrom et al. (2008) to estimate faveolar volume at depth and compared the estimated faveolar collapse depth from this model with empirical data previously published in green turtles forcibly submerged inside hyperbaric chambers (Berkson, 1967). This model assumed that the total lung capacity (TLC) includes both the maximum faveolar volume (VfA_max_) and the volume of dead space in the trachea and bronchi (VD; Fahlman et al., 2009, 2018). We obtained measurements of TLC for each species from the relevant literature (Table 2; Berkson, 1967; Table 3; Lapennas and Lutz, 1982; Lutz and Bentley, 1985; Lutcavage et al., 1990; Lutcavage and Lutz, 1991; Hochscheid et al., 2007). However, as we were only able to find measurements of VD for green turtles (Berkson, 1967; Gatz et al., 1987), we assumed that VD was equivalent (7% of TLC) in both loggerhead and leatherback turtles. We assumed that gas exchange only occurred in the faveoli, and that gas exchange stopped immediately upon faveolar collapse (i.e., when the faveolar volume was zero, VfA = 0).

**Table 2:** Estimated model variables for mass-specific total lung capacity (TLC, ml • kg^−1^), O_2_ stores (ml • kg^−1^) of the lung (l-O_2_), blood (b-O_2_), and muscle (m-O_2_), hemoglobin concentration ([Hb], g • dl^−1^), packed cell volume (PCV, %), myoglobin concentration ([Mb], g Mb • kg^−1^ muscle).

Species	TLC	l-O_2_	b-O_2_	m-O_2_	[Hb]	PCV	[Mb]	P50
Loggerhead	(113.6 *body mass*^0.923^) • *body mass*^−1^	16.4	7.84	3.93	0.088	30	2.9^*^	25
Leatherback	64	9.25	13.8	4.50	0.156	39	4.9	40
Green	115	16.6	8.72	3.93	0.098	29	2.9	47

*The hemoglobin P50 (mmHg) was selected from measurements in each species, and the Hill coefficient for the O_2_ dissociation curve was assumed to be 2.8 from measurements in the loggerhead sea turtle (**Wood et al., 1984**). (**Berkson, 1967*;*Lapennas and Lutz, 1982*;*Lutz and Bentley, 1985*;*Lutcavage et al., 1990*;*Lutcavage and Lutz, 1991*;*Hochscheid et al., 2007**).*

* No data so assumed equal to another species.

**Table 3:** Body compartment size as a percentage of body mass in loggerhead, green, and leatherback turtles combined.

Model	Blood	Brain	Fat/Bone	Central Circulation	Muscle
Control	7.0^*^	0.06	30	9.0	53.94
Obese	6	0.054	40	8	45.946
Emaciated	8	0.066	20	10	61.394

* *Blood volume assumed between the values measured in the leatherback and green turtles (Lutcavage et al., 1968, 1992)*.

To account for the relationship between pulmonary shunt and faveolar collapse, we used Eq. 4 in Fahlman et al. (2009). We also estimated the faveolar volume (Eq. 4 in Bostrom et al., 2008), using the parameters as previously defined for human alveoli (*a* = 1.04, *b* = 0.20, and *c* = 1.21). Finally, we estimated dead space in the upper airways (Eq. 5 in Bostrom et al., 2008) and applied the parameters as previously defined for a compliant human trachea (Kp = −6.44, *n* = 0.74). We used the same parameters for all three turtle species.

### Compartment Size

We generated estimates of the relative size of the four compartments in the model using computed tomography of five loggerhead turtles ranging in body mass from 3.2 to 48.7 kg (Appendix 1). To support these results, we also performed mass dissections of two loggerheads, one leatherback, and nine green turtles ranging in body mass from 1 to 355.9 kg and encompassing both sexes. Dissections were conducted by the Fundación Oceanogràfic de la Comunidad Valenciana which is registered as a research unit under the official ID number: ES460250001024 under the collaboration agreement with the Conselleria d’Agricultura, Medi Ambient, Canvi Climàtic i Desenvolupament Rural of the Valencian Regional Goverment, the National Oceanic and Atmospheric Administration (Florida Fish and Wildlife Conservation Commission Marine Turtle Permit 20–081), and North Carolina State University (North Carolina Endangered Species Permit 20ST42). Organs were removed intact and as much blood as possible drained from the tissues prior to weighing. To weigh the heart, the great vessels were severed at the pericardium. Due to the difficulties separating fat and muscle, we did not attempt to estimate the fat/bone and muscle compartments during dissections.

To estimate blood volumes, we used a value midway between those measured in leatherback and green turtles (Thorson, 1968; Lutcavage et al., 1992; Table 3). Blood was separated into arterial and venous, respectively, 33 and 67% of the total blood volume (Fahlman et al., 2006). After the arterial blood gases were exchanged with the tissue, the venous side from all compartments joined immediately to form mixed venous blood, as previously detailed (Fahlman et al., 2006, 2009).

As we observed higher variability between individuals than between species and proportions in compartment sizes among species were similar, we combined the results from all the species to characterize compartment sizes for a “generic” sea turtle (Table 3). This turtle was composed of 7.00% blood, 0.06% brain, 30.00% fat/bone, 9.00% central circulation, and 53.94% muscle.

### Cardiac Output and Blood Flow Distribution

We defined cardiac output ($\overset{\cdot }{Q_{tot}}$) at the surface (i.e., depth = 0) based on measurements from green turtles (*body mass =* 1.2 kg; West et al., 1992). To account for allometric differences between species in body mass, we used the following equation:

(1) $$s{\dot{Q}}_{tot}=\dot{Q}\;•\;\left(bodymass^{-0.25}\;•\;1.2^{-0.25}\right)$$

where 8.4 was the $\overset{\cdot }{Q_{tot}}$(l • min^−1^) and s$\overset{\cdot }{Q_{tot}}$is the mass-specific $\overset{\cdot }{Q_{tot}}$ (Davis and Kanatous, 1999). At the surface, the model transferred 60% of $\overset{\cdot }{Q_{tot}}$to the central circulation compartment, 34% to the muscle compartment, 4% to the brain compartment, and 2% to the fat and bone compartment. As no empirical data on this exist for sea turtles, we chose these values based on comparable data from marine mammal studies (Fahlman et al., 2018). While diving, the distribution of blood flow was tested iteratively to optimize O_2_ utilization and thus maximize the aerobic dive duration (Fahlman et al., 2006, 2009). This resulted in 92.8, 5.0, 2.0, and 0.2% of s$\overset{\cdot }{Q_{tot}}$ being directed to the central circulation, muscle, brain, and fat and bone, respectively, while diving.

When at the surface, we used a metabolic rate of 4.23 ml O_2_ • min^−1^ • kg^−1^ for each compartment. This is comparable to those reported in the literature for exercising leatherback, loggerhead, and green turtles on land under air temperatures between 26 and 30°C (Jackson and Prange, 1979; Butler et al., 1984; Lutcavage et al., 1987, 1990, 1992; West et al., 1992). We used values for exercising MR instead of resting MR, as cardiac output is often higher at the surface to recover from previous dives (Southwood et al., 1999). When diving, we assumed that the metabolic rate was 5% of that at the surface, i.e., 0.21 ml O_2_ • min^−1^ • kg^−1^ based on data from a leatherback turtle conducting a 34-min dive (Southwood et al., 1999). This value also lies within the range of metabolic rates reported by Jackson and Prange (1979), Butler et al. (1984), Lutcavage et al. (1987, 1990, 1992), and West et al. (1992), and it also ensured that the modeled turtles did not run out of O_2_ during long dives.

### Hemoglobin and Myoglobin Characteristics

We used published data on blood packed cell volume (PCV, %), hemoglobin ([Hb]), and muscle myoglobin ([Mb], Table 2) concentrations for each species (Lapennas and Lutz, 1982; Lutz and Bentley, 1985; Lutcavage et al., 1990). A P_50_ of the O_2_ dissociation curve for each species was gleaned from published literature (Table 3). We used a Hill coefficient of 2.7 for all species (Berkson, 1967; Wood et al., 1984; Lutcavage et al., 1990).

### Gas Dynamics Computations

We used O_2_, CO_2_, and N_2_ gas balance equations from Fahlman et al. (2006) to estimate gas transport in the lungs and tissues over short-time intervals and small blood volumes (package volume). For these equations, the partial pressure/tension of each gas was used as the driving force for gas exchange, and each compartment was modeled as a single homogenous tissue with a one-to-one perfusion rate and tissue gas solubility (Fahlman et al., 2006).

### Experiments

We ran several model variations on each of the turtle datasets to test the effects of varying: (1) body-condition and (2a) the surface s$\overset{\cdot }{Q_{tot}}$and (2b) diving s$\overset{\cdot }{Q_{tot}}$ on venous PN_2_

(1) To assess how changes in body-fat affect venous PN_2_, we varied the relative volume of the fat/bone compartment in the control model (30%) to either 20 or 40% to represent extremes of nutritional condition. When changing the relative size of the fat/bone compartment, the contribution of the other compartments was adjusted accordingly to ensure that they maintained the same overall mass (Table 3)

(2a and b) To assess the effect of varying the s$\overset{\cdot }{Q_{tot}}$, we adjusted the surface s$\overset{\cdot }{Q_{tot}}$from the control model (s$\overset{\cdot }{Q_{tot}}$of 7 ml • min^−1^ • kg^−1^) to either 2.5 ml • min^−1^ • kg^−1^ (Surface-Low) or 10 ml • min^−1^ • kg^−1^ (Surface-High). We also examined the effect of varying the s$\overset{\cdot }{Q_{tot}}$during diving from the control model (5.0% of the control s$\overset{\cdot }{Q_{tot}}$) to either 10.0% (dive-low) or 3.3% (dive-high).

Finally, to evaluate how changes in body-fat or cardiac output affected the risk of GE, we calculated the end dive PN_2_ saturation of the mixed venous blood during the last 5 s of each dive. We then measured the percentage change between the mean (over all dives) and the maximum end dive PN_2_ for the different models using the formula:

(2) $$\begin{array}{l} \mathrm{Saturation}\left(\%\right)\;=\left(\mathrm{control model}-\mathrm{model variation}\right)\\ \;\;\;\;\;\;\;\;\;\;\;\;\;\;\;\;\;\;\;\;\;\;\;\;\;\;\;\;\;\;\;\;\;\;\;\;\;\;\;\;\;\;\;•{\mathrm{control model}}^{-1}\;•\;100 \end{array}$$

### Model Validation and Analysis

While there was no published data available on arterial PN_2_ levels in free-diving sea turtles, Berkson (1967) measured arterial PN_2_ in green turtles during forced submergence. Thus, to assess the accuracy of our model for estimated arterial PN2, we compared the modeled results to those observed in Berkson (1967) during comparable dives.

As these are modeling based on the physiological assumptions from a limited number of individuals, we opted to only perform limited statistical analyses on the results. As such, these results should be considered largely descriptive and are simply to provide a framework for developing testable hypotheses in future studies.

## Results

Dive summaries for each turtle are reported in Table 4. We divided these summaries between shallow (0–30 m), medium (30–90 m), and deep (>90 m) dives. We selected these thresholds according to the values in Berkson (1967) as there should be limited impact of pressure on lung diffusion at 30 m or less and there should be almost no lung diffusion deeper than 90 m. The table includes the average (±SD) dive duration, the maximum depth reached during each dive, and the average depth for each dive (Table 4).

**Table 4:** Dive duration summaries from six leatherback, three loggerhead, and four green turtles.

		Shallow (< 30 m)			Medium (30–90 m)			Deep (> 90 m)		
Species	ID	Dive duration(s)	Mean max. Depth(m)	Proportion of dives (%)	Dive duration(s)	Mean max. Depth(m)	Proportion of dives(%)	Dive duration (s)	Mean max. Depth(m)	Proportion of dives(%)
Leatherback	Dc1	209 ± 251	6.3 ± 7.0	87.2	827 ± 157	49.1 ± 14.4	11.6	839 ± 97	108.7 ± 15.3	1.2
Leatherback	Dc2	177 ± 222	4.1 ± 5.3	90.3	1,155 ± 248	55.5 ± 18.6	6.5	1,345 ± 141	136.4 ± 88.3	3.2
Leatherback	Dc3	74 ± 138	9.5 ± 7.0	84.0	244 ± 349	51.3 ± 15.8	14.2	367 ± 473	134.1 ± 67.8	1.9
Leatherback	Dc4	175 ± 162	7.8 ± 8.0	75.0	581 ± 161	48.2 ± 13.8	23.2	812 ± 133	120.7 ± 36.9	1.8
Leatherback	Dc5	230 ± 231	9.3 ± 9.4	71.7	775 ± 234	49.5 ± 15.2	25.0	1,166 ± 211	114.3 ± 20.0	3.2
Leatherback	Dc6	156 ± 174	4.3 ± 5.5	92.8	911 ± 199	54.9 ± 15.9	6.2	1,063 ± 98	115.9 ± 38.7	0.9
Loggerhead	Cc1	150 ± 499	0.7 ± 1.9	97.3	1,396 ± 604	44.6 ± 6.5	2.7	-------	-------	-------
Loggerhead	Cc2	181 ± 396	1.1 ± 2.3	94.2	1,685 ± 840	53.3 ± 7.5	5.8	-------	-------	-------
Loggerhead	Cc3	256 ± 544	1.0 ± 2.1	93.9	2,117 ± 439	66.3 ± 8.9	6.1	-------	-------	-------
Green	Cm1	183 ± 438	2.3 ± 4.4	88.0	2,705 ± 1,215	58.1 ± 17.7	10.7	3,106 ± 891	96.9 ± 6.5	1.2
Green	Cm2	502 ± 542	5.0 ± 5.1	96.1	1,314 ± 479	48.4 ± 9.7	3.9	-------	-------	-------
Green	Cm3	324 ± 382	4.6 ± 5.0	97.6	892 ± 390	44.2 ± 11.1	2.4	944	90.5	<0.01
Green	Cm4	431 ± 500	2.4 ± 1.8	100	-------	-------	-------	-------	-------	-------

*Dives were separated by depth into shallow dives (F 30 m), medium dives (30–90 m), and deep dives (> 90 m). Within each depth category, we calculated the mean dive duration (± SD), mean maximum dive depth (m), and the proportion of dives within each dive bin (%)*.

### Model Validation

**Figure 1:**
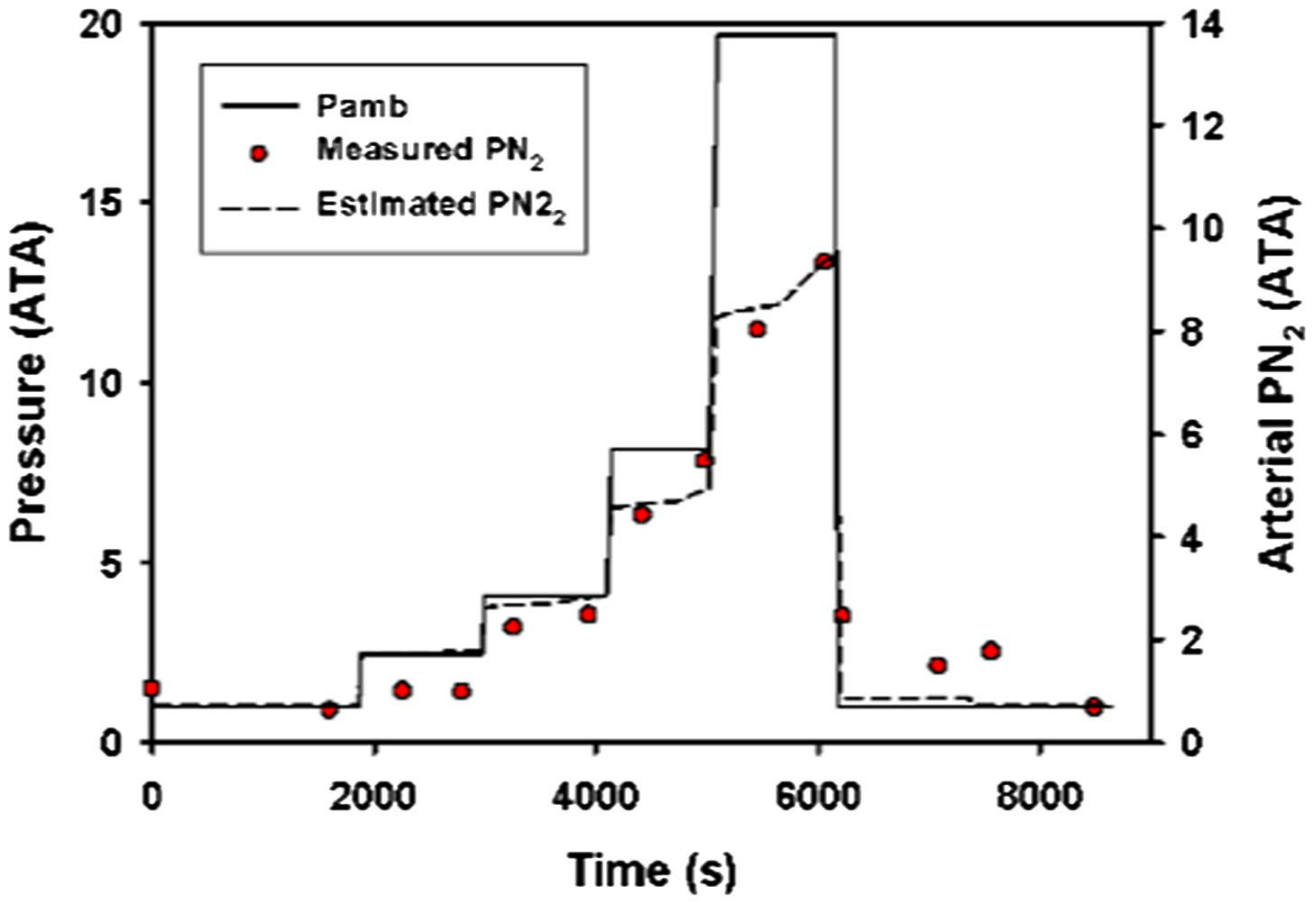
Measured (red circles) and estimated (broken black line) arterial N_2_ tension (PN_2_) in a Pacific green turtle (*Chelonia mydas agassizii*) during a step-wise forced dive to 19.4 ATA (Berkson, 1967). The model used a surface sof 3.42 ml • min^−1^ • kg^−1^ and a diving s0.17 ml • min^−1^ • kg^−1^ and a diving lung volume 50% of total lung capacity.

When comparing data on arterial PN_2_ during forced submergence from Berkson (1967) to the model output, the modeled data were never more than 1.5 ATA from the measured data (Figure 1). Furthermore, the modeled data strongly reflected the observed changes in arterial PN_2_ at different pressures.

### Control Model

**Figure 2:**
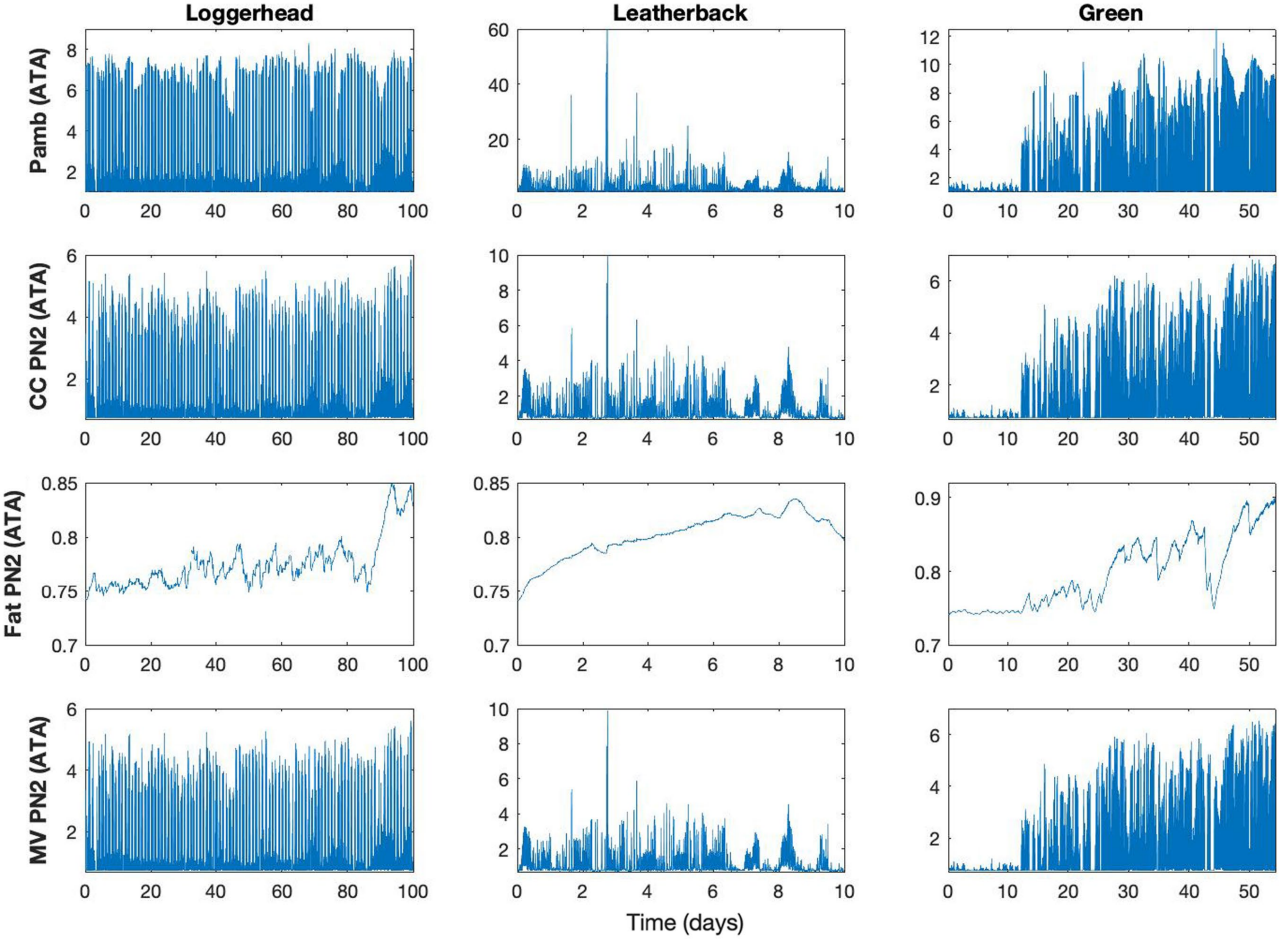
Model result for control model, showing ambient pressure (Pamb, ATA), and tissue gas tension (PN2) for central circulation (CC), fat, and mixed venous blood (MV) in a single loggerhead (Turtle ID 14293), leatherback (Turtle ID 10A1059a), and green sea turtles (Turtle ID 37800, Table 2). Data are shown for 100, 10, and 55 days for loggerhead, leatherback, and green turtles, respectively. The Pamb was used to generate the model output for the various tissues and the difference in tissue PN2 between CC (fast tissue) and fat (slow tissue), with the PN2 in MV is the composite of all tissues and their relative blood flow.

**Figure 3:**
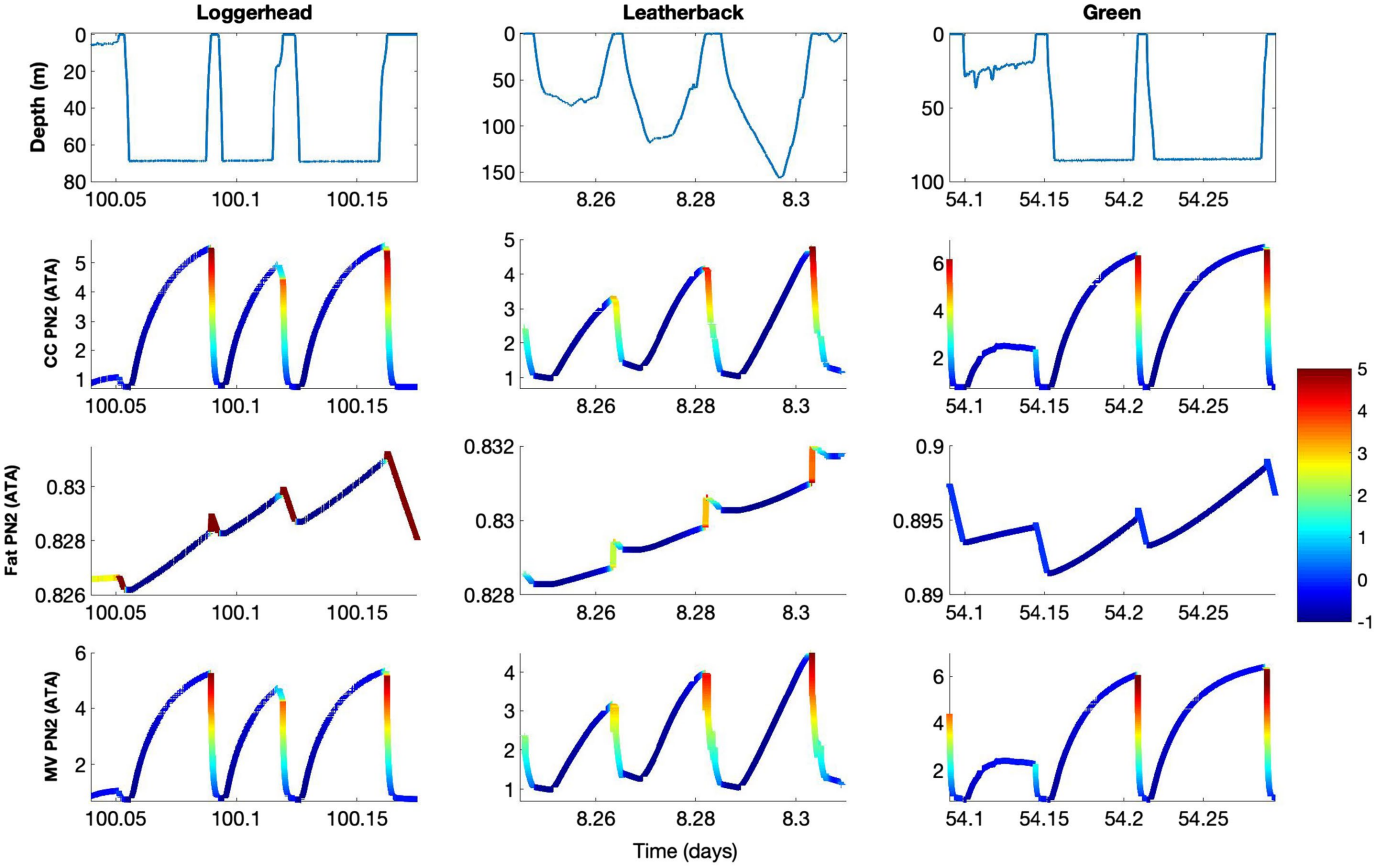
Model result for control model, showing depth (m), and tissue gas tension (PN_2_) in central circulation (CC), fat, and mixed venous blood (MV) in loggerhead (Turtle ID 14293), leatherback (Turtle ID 10A1059a), and green sea turtles (Turtle ID 37800, Table 2). The data presented are a subset of those in Figure 2 to show specific dive patterns and blood and tissue PN_2_ levels. The theoretical supersaturation [(mixed venous PN_2_ – ambient pressure) • ambient pressure^−1^, in ATA] for each of the tissues and the mixed venous blood are shown in color. The color bar shows the level of supersaturation, where negative values (blue) indicate N_2_ uptake and positive values indicates the whole body, or mixed venous PN_2_, exceeds the ambient pressure, i.e., risk of Gas Emboli formation (GE) and pathology (GEP). Thus, increasing value, i.e., yellow to red, indicates increasing risk of GEP. The results show increasing risk of GEP during ascent and how ascent rate or how sudden stops during ascent reduces risk, e.g., ascent from second dive in loggerhead or leatherback turtles.

The model output showed that the accumulation of N_2_ into the central circulation occurred faster than it did in the fat (Figure 2). Because of this, PN_2_ within the central circulation compartment tended to reflect the ambient pressure, while fat PN_2_ was more influenced by the dive profiles over a series of hours to days (Figure 3). As such, central circulation only briefly contributes to GE risk during the ascent as N_2_ was rapidly removed and the supersaturation decreased. The fat compartment, on the other hand, slowly varied with time and depended on the previous diving pattern. Furthermore, unlike the central circulation, supersaturation occured close to the surface and during the entire surface interval. Nevertheless, the overall fat PN_2_ saturation seldom exceeded 1 ATA and therefore does not contribute appreciably to GE risk.

Using the control model and combining the results from all three species, mean end-dive PN_2_ increased with depth for central circulation (χ^2^ = 32, 2 df, *p* < 0.01), muscle (χ^2^ = 22, 2 df, p < 0.01), brain (χ^2^ = 20, 2 df, *p* < 0.01), and mixed venous (χ^2^ = 34, 2 df, *p* < 0.01) but not for the fat compartment (χ^2^ = 2.4, 2 df, *p* > 0.2). When comparing dives within the shallowest dive bin (<30 m) to the deepest dive bin (>90 m), mean end-dive PN_2_ increased by 245% for central circulation, 11% for muscle, 189% for brain, and 221% for the mixed venous compartment. When the three species were considered separately, leatherback turtles had the lowest mean end-dive PN_2_, with green turtles having intermediate values, and loggerhead turtles having the highest (χ^2^ = 8.49, 2 df, *p* = 0.014; End-dive PN2 = 2.73 (±0.31)−0.56 (±0.28) × leatherback + 0.54 (±0.41) × loggerhead−0.63 (±0.30) medium−1.71 (±0.29) shallow).

With data from all three species combined, maximum end-dive PN_2_ increased with depth for central circulation (χ^2^ = 11.5, 2 df, *p* < 0.01), brain (χ^2^ = 8.8, 2 df, *p* = 0.012), and mixed venous blood (χ^2^ = 12.1, 2 df, *p* < 0.01), but decreased for fat (χ^2^ = 7.9, 2 df, *p* = 0.019), and did not change for muscle (χ^2^ = 5.1, 2 df, *p* = 0.079). Comparing dives in the shallow depth range to those in the deep depth bin, maximum end-dive PN_2_ increased by 80% for central circulation, by 68% for brain, and by 81% for mixed venous blood. For brain, both depth range and species helped to explain the variation (χ^2^ = 7.39, 2 df, *p* = 0.025). Overall, mean end-dive PN_2_ for brain increased with depth and leatherback turtles had the lowest maximum end-dive PN_2_, while green turtles had intermediate values, and loggerhead turtles had the highest (change in end-dive brain PN_2_ (%) = 4.23 (±0.65) − 0.50 (±0.66) × leatherback + 1.95 (±0.92) × loggerhead – 1.03 (±0.54) medium −1.86 (±0.53)). The maximum end-dive PN2 for mixed venous blood was 9.40 ATA, 6.17 ATA, and 5.03 ATA for leatherback, green, and loggerhead turtles, respectively, representing supersaturation ratios [(mixed venous PN_2_ – ambient pressure) • ambient pressure^−1^, or M-ratios] of 12.7, 8.3, and 6.8.

### Model Variation – Body-Fat

**Figure 4:**
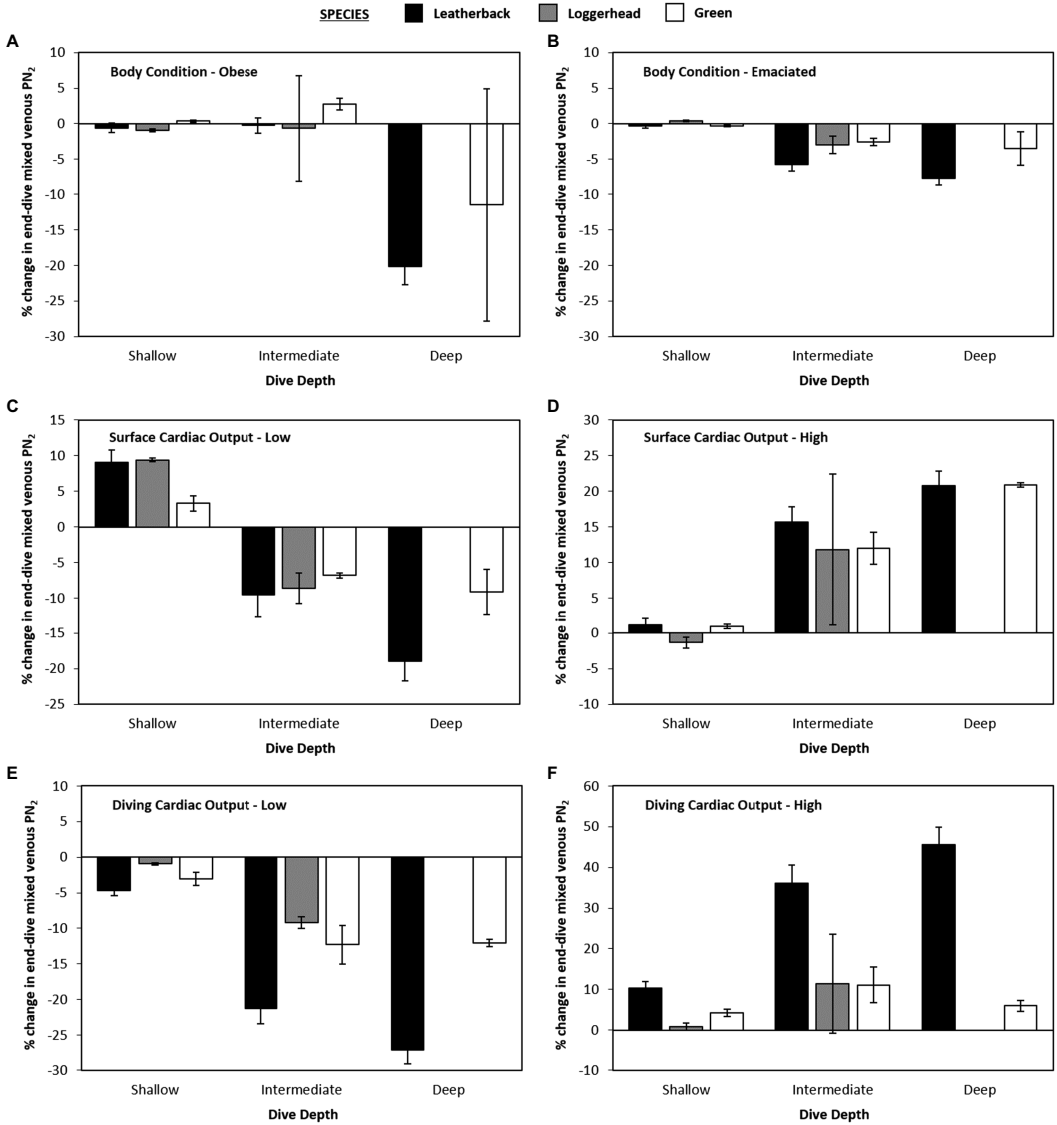
Data showing percent changes in mean end-dive PN_2_ for variation in gas dynamics model as compared with the control model [(new model – control model) • control model–1 • 100].

**Figure 5:**
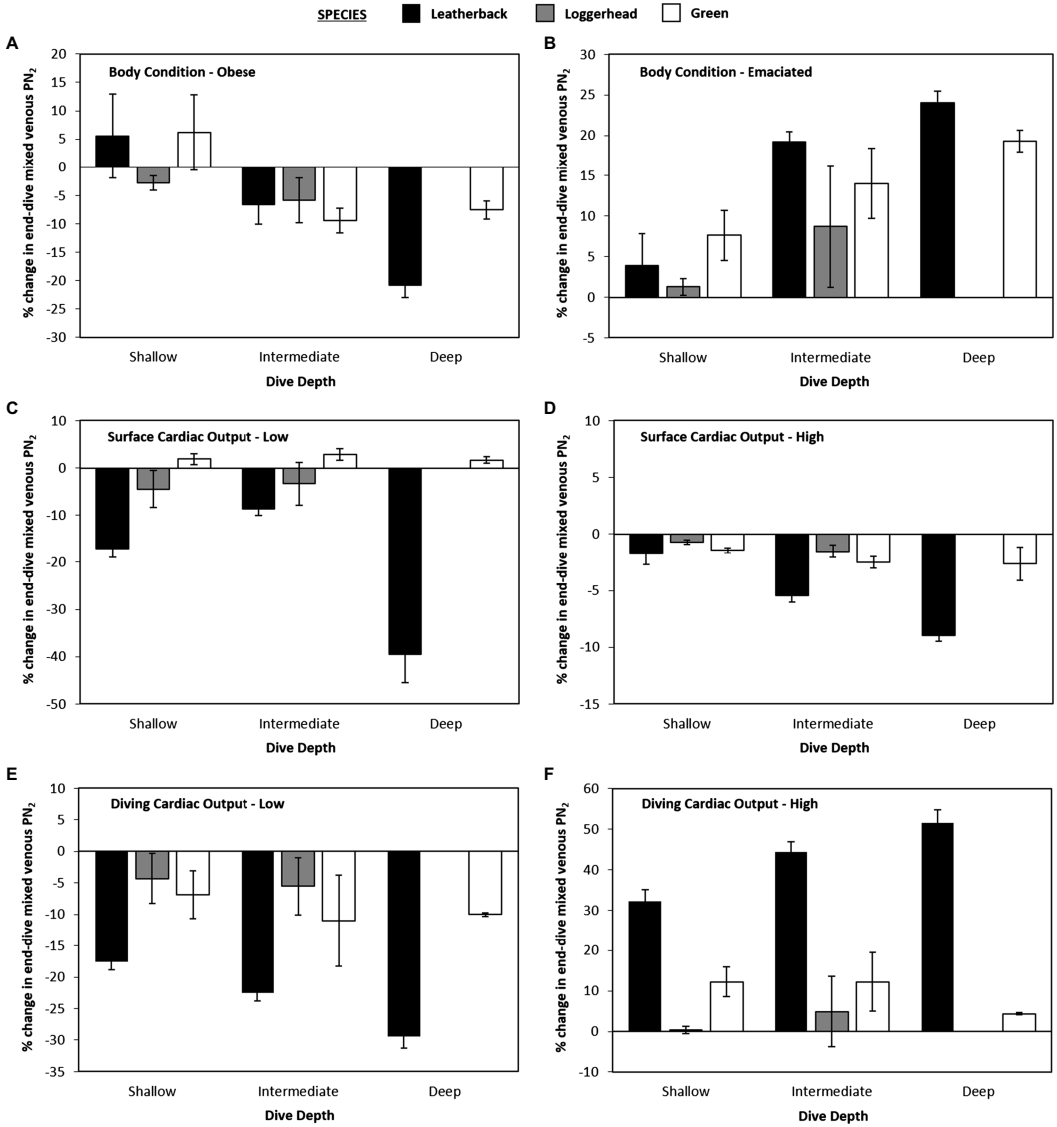
Data showing percent changes in maximal end-dive PN_2_ for variation in gas dynamics model as compared with the control model [(new model – control model) • control model–1 • 100].

When body fat was increased from 30 to 40% of body mass or decreased from 30 to 20% of body mass, there was no effect observed on mean or maximum end-dive mixed venous PN_2_ for either shallow or intermediate depths when all three species were combined (Figure 5). However, for the deepest depth bin, increasing or decreasing body-fat reduced the mean end-dive mixed venous PN_2_ (χ^2^ > 10.0, 2 df, *p* < 0.01; Figures 4A,B) and the maximum end-dive mixed venous PN_2_ (Figures 5A,B). Thus, a model that included depth, species, and body-fat was warranted; % change in maximal end-dive mixed venous PN_2_ = −13.3 (±1.2) − 12.4 (±2.6) leatherback – 5.2 (±3.8) logger + 12.5 (±3.0) medium depth + 11.0 (±2.9) shallow depth + 8.6 (±2.3) emaciated (χ^2^ > 14.4, 1 df, *p* < 0.01).

### Model Variation – Cardiac Output at Surface

When s$\overset{\cdot }{Q_{tot}}$was either increased or decreased, there were no consistent differences in mean mixed venous end-dive PN_2_ among species at the shallow depth bin (*p* > 0.3 for all, Figures 4C,D). For both medium and deep depths, when s$\overset{\cdot }{Q_{tot}}$was increased to 10 ml • min^−1^ • kg^−1^ (Surface-High) this increased mean end-dive PN_2_ by 10.6% (Figure 4D), while a decrease in s$\overset{\cdot }{Q_{tot}}$to 2.5 ml • min^−1^ • kg^−1^ (Surface-Low; Figure 4C) caused a 4.5% decrease in mean end-dive PN_2_ [χ^2^ > 26.5, 1 df, *p* < 0.01, change in end-dive PN_2_ (%) = 10.6 (±1.9) – 15.1 (±2.7) × (Surface-Low)].

For maximum end-dive mixed venous PN_2_, the changes were similar, with 43% increase in surface s$\overset{\cdot }{Q_{tot}}$(Surface-High) causing an increase in maximum end-dive PN_2_ by 13.4% (Figure 5D), and 64% decrease (Surface-Low) in s$\overset{\cdot }{Q_{tot}}$causing a 5.4% decrease [Figure 5C, χ^2^ > 31.0, 1 df, *p* < 0.01, change in end-dive PN_2_ (%) = 13.4(±2.1) – 18.7 (±3.0) × (Surface-Low)].

### Model Variation – Cardiac Output at Depth

When altering diving s$\overset{\cdot }{Q_{tot}}$, there was no statistically significant effect of dive depth on mean end-dive mixed venous PN_2_ when all three species were combined (*p* > 0.5 for all, Figures 4E,F). Considering all species and depths together, an increase in diving s$\overset{\cdot }{Q_{tot}}$ from 5 to 10% lead to 20.6% increase in mean end-dive mixed venous PN_2_ (Figure 5E)_._ A decrease in diving s$\overset{\cdot }{Q_{tot}}$from 5 to 3.3% (Figure 4F) lead to a decrease of 13.2% in the mean end-dive mixed venous PN_2_ [χ^2^ > 52.8, 1 df, *p* < 0.01, change in end-dive PN_2_ (%) = 20.6(±2.7) – 33.9 (±3.8) × (1/30)].

When varying diving s$\overset{\cdot }{Q_{tot}}$, there was uniform effect on the maximal end-dive PN_2_, however, there were differences between species. Specifically, the maximum end-dive mixed venous PN_2_ was higher for leatherback turtles than either loggerhead or green turtles at all depths. In addition, the lower diving s$\overset{\cdot }{Q_{tot}}$significantly reduced the maximal end-dive PN_2_ (Figures 5E,F); [χ^2^ > 6.21, 1 df, *p* = 0.013, change in maximal end-dive PN_2_ (%) = 32.2 (±3.1) – 44.9 (±3.8) × (1/30) – 9.6 (±3.8) loggerhead/green].

## Discussion

GE associated with fisheries bycatch and other submerged gear interactions could be a significant threat to sea turtles worldwide (García-Párraga et al., 2014; Fahlman et al., 2017; Crespo-Picazo et al., 2020; Harms et al., 2020; Parga et al., 2020). To better understand how sea turtles manage the risk of GE during natural diving behavior, we adapted a theoretical model to estimate blood and tissue N_2_ tensions in diving marine mammals to fit loggerhead, leatherback, and green sea turtles. We also applied the new model to assess how body-fat and cardiac output influence the potential for GE during normal diving behavior in sea turtles. Demonstrating the accuracy of the model, it provided a reasonable estimate for blood and tissue PN_2_ from previously conducted forced diving experiments (Berkson, 1967). By varying the model, we observed that higher fat reserves can reduce end-dive PN_2_ by up to 25%. We also observed that an increase in surface s$\overset{\cdot }{Q_{tot}}$, as likely occurs as the turtle reaches the surface to help to increase O_2_ uptake and CO_2_ removal (Okuyama et al., 2020), led to an increase in end-dive PN_2_. Finally, the model showed that leatherback turtles have lower mean end-dive blood and tissue PN_2_, but higher maximum end-dive values, than loggerhead or green turtles during routine diving behavior. While higher maximum end-dives values may be expected considering that leatherback turtles dive considerably deeper than the other two species (Robinson and Paladino, 2015), the fact that they had lower mean end-dive blood and tissue PN_2_ also suggests that their increased body-size helps to reduce the risk of GE.

### Model Assumptions and Limitations

Our goal was to construct the initial framework for a model to understand gas dynamics in diving sea turtles. As no such models previously existed, a logical first step was to adapt a previously constructed and validated model for another air-breathing vertebrate. While we chose to adapt a model originally designed for marine mammals (Fahlman et al., 2006, 2009, 2021), there are several key anatomical and physiological differences between marine mammals and sea turtles that have not yet been incorporated into the new model. Firstly, and of considerable importance, is that sea turtles, unlike marine mammals, are at least partially ectotherms. Specifically, even though several studies have shown that sea turtles can maintain body-temperature elevated above ambient conditions (Paladino et al., 1990; Sato et al., 1995), their body temperatures still fluctuate over wider ranges than marine mammals. While we attempted to control for this by only analyzing diving data for sea turtles within a restricted range of sea surface temperatures (19–29°C), the effect of temperature on both the metabolic rate and the gas tensions/solubilities cannot be ignored (Lutz et al., 1989).

Another important difference between marine mammals and sea turtles is that sea turtles have muscular sphincters within their pulmonary arteries and a partially compartmentalized ventricle that allow for central intracardiac shunting (García-Párraga et al., 2018a; Burggren et al., 2020). These features could allow a complete shunt and cessation of gas exchange as the animal begins the dive. In fact, this could be the mechanism by which sea turtles avoid GE (García-Párraga et al., 2018b; Fahlman et al., 2021). In this model, we assumed that the pulmonary shunt develops due to the passive compression of the terminal air spaces and the structural properties of the conducting airways and faveoli (Bostrom et al., 2008; Fahlman et al., 2009). While this is not necessarily the case in reptiles, the exact factors influencing the cardiac shunt in sea turtles are not well-established (Burggren et al., 2020). Without having a solid understanding of the functioning of the cardiac shunt, we adjusted the model so that the arterial PN_2_ reflected those of forced-diving sea turtles in Berkson (1967). This meant that the physiological shunt and the potential anatomical shunt were expressed together as a pulmonary physiological shunt. In addition, we acknowledge that forced-diving experiments in Berkson (1967) would likely elevate blood and tissue N_2_ levels beyond normal values, and our model likely thus provides a conservative estimate of blood and tissue N_2_ levels. However, if we consider exercise as one of the main driving forces in the cardiac output adjustment (Okuyama et al., 2020), physically restrained animals could minimize N_2_ uptake reflecting lower N_2_ values compared to free swimming or entrapped individuals with restricted movement.

Other significant assumptions with the model resulted from differences between the datasets available for each species. For example, we utilized diving data from subadult loggerhead in foraging habitats, while the data for leatherback and green turtles were from adult females in inter-nesting habitats. Indeed, it is known that foraging and inter-nesting turtles exhibit differing dive behaviors (Robinson and Paladino, 2015). Other factors including life-stage and temperature are also known to influence diving behavior but are once again not standardized between our turtle diving datasets.

As is the case for all theoretical physiological models, the complexity of the entire system cannot be incorporated holistically into a mathematical model. While such arguments are often used to disparage the value of such exercises, we stress that we aimed to build the foundation upon which more detailed assessments of how factors, such as behavior, life-stage, or life-stage, can influence the risk of GE in sea turtles. As such, the goal of this exercise was to try and to identify which factors and assumptions need to be prioritized in future models to gain a more accurate understanding of gas dynamics in sea turtles.

### General Patterns in PN_2_ and Inter-Specific Variation

In the basic model, the mean end-dive PN_2_ differed between species and maximum depth of the previous dive. For shallow and deep dives, the end-dive PN_2_ values were, respectively, between 38–111% and 193–342% higher as compared with the surface equilibrium PN_2_. Interestingly, maximum PN_2_ increased with dive depth in all turtle species. This suggests that the risk of GE increases with depth as observed in previous studies (Fahlman et al., 2017).

### Effect of Body-Fat on Gas Emboli Formation

While we observed no effect of body-fat on end-dive mixed venous PN_2_ at depths less than 90 m, significant differences were observed beyond 90 m. Interestingly, end-dive mixed venous PN_2_ was highest at intermediate body-fat levels, and it dropped if body-fat was either increased to 40% or dropped to 20%. Moreover, these differences were up to 25% lower in leatherback turtles relative to loggerhead or green turtles. These findings suggest that the higher levels of fat in leatherback turtles could perhaps reduce the effects of GE and play a role in the ability of leatherbacks to dive several times deeper than any other sea turtle species (Robinson and Paladino, 2015). That said it is unclear why lower body-fat values also result in reduced end dive PN_2_.

### Effect of Cardiac Output on Gas Emboli Formation

Past studies have shown that variation in blood flow, through changes in cardiac output, and the level of gas exchange are the physiological variables that have the greatest effect on N_2_ uptake and removal (Fahlman et al., 2006, 2009; Hooker et al., 2009). Our model provided evidence that this may also be the case for sea turtles as a reduction of the diving s$\overset{\cdot }{Q_{tot}}$ to 3.3% caused a mean 13.2% decrease in end-dive PN_2_, while increasing the diving s$\overset{\cdot }{Q_{tot}}$ to 10% caused a mean 20.6% increase in end-dive PN_2_. Similar patterns were also observed for maximum end-dive PN_2_. Nevertheless, these patterns differed by species and leatherback turtles exhibited lower mean end-dive PN_2_ at both higher and lower diving s$\overset{\cdot }{Q_{tot}}$.

### Implications for Gas Embolism Formation

Our model suggests that during “natural” diving behavior sea turtles experience blood and tissue N_2_ levels that would cause decompression sickness in land mammals of similar size. Indeed, the maximal end-dive PN_2_ values between species ranged from 5.03 ATA to 9.40 ATA, which is considerably higher than the level of end-dive PN_2_ that would result in severe DCS in 50% of similarly sized humans (Fahlman et al., 2020). As it is highly unlikely that sea turtles are perpetually suffering from GE during routine diving behavior, we propose that turtles, much like marine mammals, must have additional behavioral, anatomical, and/or physiological mechanisms to reduce N_2_ uptake that are not currently considered in this model (García-Párraga et al., 2018a; Burggren et al., 2020; Fahlman et al., 2021).

One mechanism that sea turtles may employ to lower the risk of GE is the Selective Gas Exchange hypothesis (García-Párraga et al., 2018b; Fahlman et al., 2021). This hypothesis, which is supported by theoretical, anatomical, and physiological studies (Fahlman et al., 2009, 2018, 2020; Olson et al., 2010; Hodanbosi et al., 2016; García-Párraga et al., 2018a), suggests that if ventilation and perfusion in the lung can be managed, it would allow exchange of O_2_ and CO_2_, with little or no exchange of N_2_ (García-Párraga et al., 2018b; Fahlman et al., 2021). In support of this, it has been shown that the pulmonary arterial sphincters contract and relax when exposed to parasympathetic and sympathetic neurotransmitters, respectively (García-Párraga et al., 2018a). If sea turtles exhibit Selective Gas Exchange, this would help to significantly reduce our modeled blood and tissue N_2_ levels and help to explain how turtles avoid GE during natural dives. It would also explain how the sympathetic stress response could result in elevated pulmonary blood flow to the still ventilated lung, yielding excessive N_2_ uptake and GE (Fahlman et al., 2021).

It is also possible that turtles couple physiological and behavioral mechanisms to further reduce the risk of GE. Indeed, sea turtles increase their heart rate, and likely cardiac output, during the ascent phase (Okuyama et al., 2020). In addition, several breath-hold diving vertebrates, including sea turtles, the beluga (*Delphinapterus leucas*; Martin and Smith, 1992), beaked whales (*Hyperoodon ampullatus*; Hooker and Baird, 1999), macaroni penguin (*Eudyptes chrysolophus*; Sato et al., 2004), and king penguins (*Aptenodytes patagonicus*; Sato et al., 2004) have been shown to reduce the ascent rate while approaching the surface. It has been suggested that this reduction may be an adaptation to reduce end-dive PN_2_ before reaching the surface (Fahlman et al., 2006; Fossette et al., 2010). When the reduction in ascent rate is coupled with increases in heart rate during the ascent, it may reduce end-dive PN_2_ by as much as 45% in marine mammals (Fahlman et al., 2006). A similar reduction in the supersaturation was seen in all the turtles in this study (Figure 4). It also appears that the tendency of sea turtles to conduct a short “pause” in their accent around 10–20 m, which we observed in all three species, may also help to reduce the supersaturation as the turtle returned to the surface (Fossette et al., 2010).

These adaptations to reduce PN_2_ during normal dives could also explain why sea turtles are predisposed to GE when caught by fisheries. For example, rapid accent to the surface in a fishing net coupled with disruption of the normal dive profile and physiological response to underwater entrapment may alter cardiac output and shunting, leading to rapid GE. It also suggests that slower retrieval of the fishing nets or trawls may help to significantly reduce blood and tissue N_2_ levels in bycatch sea turtles. If operationally possible, this may provide a simple and cost-effective strategy to minimize GE in turtle by-catch and could have the added benefit of reducing fish by-catch as well (Grothues et al., 2017). However, a significant concern with longer ascent times is increased risk of drowning, which may negate any benefits.

### Future Directions

Our model provides a framework for future studies on the analysis of GE in sea turtles. While we adapted the model to test the effects of body-fat and cardiac output on GE, this model could be adapted in other beneficial ways. Indeed, incorporating a simulated capture event, whereby the speed of the ascent to the surface or the total time spent at depth is varied. The model could also be adapted to assess how variation in the cardiac shunt plays a role in GE during both routine dives and during incidental capture. The strong effect of cardiac output on GE also highlights that more information is needed on cardiac dynamics in diving sea turtles.

## Data Availability Statement

The raw data supporting the conclusions of this article will be made available by the authors, without undue reservation.

## Ethics Statement

The animal study was reviewed and approved by Purdue Animal Care and Use Committee, NOAA Animal Care and Use Committee.

## Author Contributions

NR and AF designed the experiment with input from BS. NR and AF wrote the manuscript with input from all authors. AF built and ran the model. NR, GB, CC-B, HH, and SP provided the data from the data loggers. BS, DG-P, and AC supervised necropsies and computed tomography. All authors contributed to the article and approved the submitted version.

## Conflict of Interest

AF was employed without salary by the company Global Diving Research Inc.

The remaining authors declare that the research was conducted in the absence of any commercial or financial relationships that could be construed as a potential conflict of interest.

## Publisher’s Note

All claims expressed in this article are solely those of the authors and do not necessarily represent those of their affiliated organizations, or those of the publisher, the editors and the reviewers. Any product that may be evaluated in this article, or claim that may be made by its manufacturer, is not guaranteed or endorsed by the publisher.

## Appendix 1

Relative compartment size (blood, brain, fat, central circulation, and muscle) from seven loggerhead, eight green, and one leatherback turtles. Compartment size in five of the seven loggerhead turtles was assessed using computed tomography, while it was measured in-situ *via* necropsy in the remaining animals. ^*^Blood was not measured directly and was estimated from values in the published literature.

**Table:**

				Compartment size (% of total body mass)				
Species	Body mass (kg)	Sex	Method for estimating compartment mass	Blood^*^	Brain	Fat/Bone	Central circulation	Muscle
Cc	3.2	Unknown	Computed tomography	6.7	<0.1	15.9	23.2	58.4
Cc	4.7	Unknown	Computed tomography	6.7	<0.1	20.2	29.7	48.0
Cc	20.0	Unknown	Computed tomography	6.7	<0.1	28.9	18.7	53.8
Cc	20.7	Unknown	Computed tomography	6.7	<0.1	32.2	17.2	48.4
Cc	48.7	Unknown	Computed tomography	6.7	<0.1	28.7	26.2	39.0
Cc	95.9	Female	Necropsy	6.7	<0.1	Not measured	12.2	Not measured
Cm	1.9	Female	Necropsy	6.7	0.1	Not measured	11.0	Not measured
Cm	4.0	Female	Necropsy	6.7	0.1	Not measured	10.0	Not measured
Cm	2.7	Female	Necropsy	6.7	0.1	Not measured	8.9	Not measured
Cm	2.6	Male	Necropsy	6.7	0.1	Not measured	8.9	Not measured
Cm	117.2	Female	Necropsy	6.7	<0.1	Not measured	8.3	Not measured
Cm	139.6	Female	Necropsy	6.7	<0.1	Not measured	9.8	Not measured
Cm	150.5	Female	Necropsy	6.7	<0.1	Not measured	9.2	Not measured
Cm	2.0	Unknown	Necropsy	6.7	0.1	Not measured	10.2	Not measured
Cm	2.2	Unknown	Necropsy	6.7	0.1	Not measured	13.2	Not measured
Dc	355.9	Female	Necropsy	6.7	<0.1	Not measured	5.0	Not measured

## Abbreviations

ATA: Atmospheres Absolute
DVfA: Faveolar Volume
GE: Gas Embolism
Mb: Myoglobin
Pamb: Ambient Partial Pressure
PN_2_: Partial Pressure of N_2_
PO_2_: Partial Pressure of O_2_
PCO_2_: Partial Pressure of CO_2_
$\overset{\cdot }{Q_{tot}}$: Cardiac Output
TDR: Time-Depth Recorder
TLC: Total Lung Capacity
VD: Volume of Dead Space in the Trachea and Bronchi
VfAmax: Maximum Faveolar Volume
s$\overset{\cdot }{Q_{tot}}$: Mass-Specific Cardiac Output
